# Minocycline prevents and repairs the skin disorder associated with afatinib, one of the epidermal growth factor receptor-tyrosine kinase inhibitors for non-small cell lung cancer

**DOI:** 10.1186/s12885-020-06797-2

**Published:** 2020-04-06

**Authors:** Kazumi Sano, Kazuhiko Nakadate, Kazuhiko Hanada

**Affiliations:** 1 Department of Pharmacometrics and Pharmacokinetics, Meiji Pharmaceutical University, 2-522-1 Noshio, Kiyose-shi, Tokyo, 204-8588 Japan; 2 Department of Basic Biology, Educational and Research Center for Pharmacy, Meiji Pharmaceutical University, 2-522-1 Noshio, Kiyose-shi, Tokyo, 204-8588 Japan

**Keywords:** Minocycline application, Improvement skin disorder, Afatinib, EGFR, NSCLC

## Abstract

**Background:**

While epidermal growth factor receptor (EGFR)-tyrosine kinase inhibitors (TKIs) exert a breakthrough effect, the incidence of skin disorders as a side effect has significantly reduced patients’ quality of life. This study aimed to develop a treatment for inflammatory ulcers as one of the side effects of afatinib (Giotrif®), a second-generation EGFR-TKI, and established a skin disorder mouse model to investigate the protective effect of minocycline.

**Methods:**

First, under inhalation anesthesia with isoflurane, the back of a male ddy mouse was shaved, and afatinib petrolatum was applied alone or in combination with minocycline to observe the state of the skin and measure transepidermal water transpiration (TEWL). Next, afatinib was administered orally to mice, and minocycline petrolatum was applied to observe whether the skin disorder was prevented and its effect on repair of the skin disorder.

**Results:**

Skin injury occurred on the back of the mouse following afatinib (1 mg/g in petrolatum) application, and scab formation was observed. Application of minocycline prevented and improved the skin disorder caused by afatinib. When the minocycline-petrolatum mixture was applied to the mouse that developed the skin disorder, a significant improvement in TEWL was observed, and skin repair was observed macroscopically.

**Conclusions:**

These results suggest that minocycline petrolatum applied locally prevents and repairs afatinib-induced skin disorders of non-small cell lung cancer patients. Histological examination of skin has provided insights into the mechanism of the occurrence of afatinib-related skin disorder and suggested the efficacy of minocycline topical application in clinical practice.

## Background

Afatinib (Giotrif®, Boehringer Ingelheim Japan, Tokyo, Japan) is one of the second-generation epidermal growth factor receptor (EGFR)-tyrosine kinase inhibitors (TKIs), an orally irreversible ErbB family blocker that covalently binds to the kinase domains of EGFR, human EGFRs (HER)2 and 4, and is clinically effective in non-small cell lung cancer (NSCLC) [1].

The skin disorders caused by afatinib are common to all EGFR-TKIs and anti-EGFR antibody drugs in general [2]. Since the incidence of skin disorders is high, and the quality of life (QOL) of patients is decreased, immediate elucidation of the pathogenesis of skin disorders and treatment based on the principle of direct approach to the skin is needed. In the last 5 years or so, supportive treatment regimens with TKIs have incorporated oral administration of minocycline for the purpose of preventing rashes [3–6]. However, this method of oral administration of minocycline is a provisional procedure similar to the treatment of acne rash in adolescents, and there is no clear evidence that oral administration of minocycline has any effect on EGFR-TKI-induced eruptions.

Minocycline is an antibiotic derived from semi-synthetic tetracycline and has a broad antibacterial spectrum from gram-positive with staphylococci, streptococci and pneumococci to gram-negative bacteria with *E. coli*. It is also approved by US Food and Drug Administration (FDA) for the treatment of some sexually transmitted disease such as chlamydia. Oral minocycline is used to treat inflammatory acne because of its antibacterial activity against *Propionibacterium acnes* and its anti-inflammatory action. These anti-inflammatory, anti-apoptotic, and antioxidant effects of minocycline have recently attracted attention [7, 8].

Torigoe et al. reported that intrathecally administered minocycline acts on microglia to suppress the itching of atopic dermatitis and improve dermatitis in atopic dermatitis model mice [9]. In addition, it has been reported that minocycline acts on one mitochondrial protein and is involved in the prevention of Parkinson’s disease (PD) onset [10]. Furthermore, minocycline has been attracting attention for its action on nerve cells, with the expectation that it could suppress the risk of developing multiple sclerosis [11].

The drug-induced skin disorders of EGFR-TKIs are side effects caused by TKI inhibiting EGFR localized in the skin. We considered that it would be appropriate to treat the adverse events at the site of expression without undue burden on the visceral system and devised a means of direct application of minocycline to the skin. For patients taking an EGFR-TKI such as afatinib, the development of a skin rash must be suppressed by prophylactic use of minocycline topical medications, and clinical use must be achieved rapidly. However, in Japan, minocycline ointment is approved for dental preparations only and cannot be applied directly to skin diseases. The novelty of our manuscript is to demonstrate that minocycline as an ointment has hidden pharmacological effects that improve the physiological environment of the skin. And the ultimate our purpose is to clarify how oral EGFR inhibitors are excreted into the skin and how they cause skin damage.

In this study, the effects of minocycline ointment on the skin damage caused by afatinib were examined in normal mice, and the conditions necessary for developing an external-use formulation were further examined.

## Methods

### Animals

Male ddy mice (5 weeks old; Japan SLC, Inc., Shizuoka, Japan) were maintained in the experimental animal facility of Meiji Pharmaceutical University. All mice were housed under standard conditions (23 ± 2 °C) with a 12:12-h light/dark cycle (lights off at 19:00). Food and water were provided ad libitum. After completion of relevant experiments, mice were euthanized by drawing blood and exsanguination from the descending aorta under isoflurane inhalation anesthesia. All procedures were approved by the Animal Care and Use Committee at Meiji Pharmaceutical University and conducted strictly in accordance with the National Institutes of Health guidelines.

### Materials

Giotrif® tablets (afatinib maleate) were obtained from Boehringer Ingelheim Japan (Tokyo, Japan). Standard material for afatinib was obtained from SYNkinase (Melbourne, Australia). Minocycline hydrochloride was obtained from Sigma Aldrich (St. Louis, MO). White petrolatum (WP) was obtained from KENEI Pharm. Co., Ltd. (Osaka, Japan). Ammonium acetate was obtained from Nacalai Tesque, Inc. (Kyoto, Japan). Liquid chromatography-mass spectrometry (LC/MS) grade acetonitrile and deionized water were obtained from Wako Chemical Industry (Tokyo, Japan). All other chemicals were of analytical grade.

### Evidence of afatinib-induced dermatitis in a mouse model

Twenty Mice were divided into five groups: group 1, control (*n* = 4); group 2 as low concentration, 0.075% afatinib application (*n* = 4); group 3, minocycline treatment after onset of the low conc. Afatinib-induced rash (*n* = 4); group 4 as high conc., 0.10% afatinib application (*n* = 4); and group 5, minocycline treatment after onset of the high conc. Afatinib-induced rash (n = 4). The number of animals used in all experiments was as small as possible to produce valid results.

#### Preparation of the skin disorder mouse model by afatinib application

A skin disorder was induced by application of 0.075% or 0.10% (0.75 or 1.0 mg/g) afatinib petrolatum ointment (0.1 g/body respectively) as group 1, 2 and 4. Observation was continued for 7 days under afatinib application.

#### Treatment of the skin disorder by simultaneous minocycline application

To estimate the concentration ratio between afatinib and 0.03% minocycline ointment (0.1 g/body), minocycline application started simultaneously with afatinib application as group 3 and 5.

### Effects of minocycline ointment on oral afatinib administrations

Mice were divided into four groups (*n* = 4/group): group 1, as control that mice received afatinib administration (20 mg/kg BW) at once a day for 18 days with vehicle (WP) pretreatment started 3 days before afatinib administration (*n* = 4); group 2, afatinib administration (20 mg/kg BW) with minocycline WP pretreatment (*n* = 4); group 3, as control that mice received afatinib administration (20 mg/kg BW) at once a day for 18 days with control treatment with vehicle (WP) after the afatinib-induced rash onset (*n* = 4); and group 4, 0.03% minocycline WP treatment after the afatinib-induced rash onset (*n* = 4). The number of animals used in all experiments was as small as possible to produce valid results.

Based on a result of the oral dose determination experiment, mice received around 0.6 mg/0.6 mL/30 g BW afatinib in 0.1% Tween 80 (20 mg/kg BW). For pretreatment of afatinib-induced skin disorders, application of vehicle (WP) or 0.03% (0.3 mg/g) minocycline WP ointment (0.1 g/body) was started 3 days before afatinib administration as group 1 and 2. On the other hand, vehicle (WP) or 0.03% minocycline application for the afatinib-induced rash started after the onset of the rash, about 10 days after oral administration as group 3 and 4.

### Measurement of transepidermal water loss (TEWL)

TEWL was measured under isoflurane anesthesia on the dorsal skin lesion three times per week using a Tewameter® T210 (Courage + Khazaka Electronic GmbH, Cologne, Germany), and the median value of three measurements at each time point was recorded.

### Tissue preparation and treatment of euthanasia

All mice were anesthetized with isoflurane (4% induction, 2% maintenance). Under inhalation anesthesia with isoflurane whole blood (ca. 1 mL) was collected from the descending aorta and then exsanguinated. Their dorsal skin lesions were quickly removed, washed with saline and stored frozen. The wet volumes of their dorsal skin samples were weighed. For histological analysis using light microscopy, a section of the dorsal skin was immersed in fixative containing 10% paraformaldehyde. After fixation, the dorsal skin was trimmed, washed with PB, dehydrated through graded concentrations of ethanol, cleared in xylene, and embedded in paraffin. Trough the above process, it is considered that mice could be euthanized by collecting whole body blood under the same effect as exsanguination under isoflurane inhalation anesthesia.

### Histological analysis

For hematoxylin-eosin (HE) staining and to further investigate skin damage status, the dorsal skin blocks were cut into 5-μm-thick sections on a microtome (REM-710; Yamato, Japan). The sections were mounted on glass slides, deparaffinized with xylene, and immersed in degraded concentrations of ethanol. After washing in distilled water, several sections from each group were stained with HE solution. After washing, the sections were dehydrated through graded concentrations of ethanol, cleared with xylene, and cover slipped. The EGFR of keratinocytes in the dorsal skin samples from each group was incubated with anti-EGFR rabbit antibody (HPA018530, Atras Antibodies AB, Bromma, Sweden). After several washings, the sections were incubated with HRP-conjugated anti-rabbit IgG antibody produced in goat (A6454, Sigma, St. Louis, MO). After the DAB reaction procedures, the sections were cover slipped. All images of stained sections were captured using a CCD camera system (BZ-X700; Keyence, Japan) [12].

### Chromatography conditions

Samples were analyzed using a Shimadzu LC/MS/MS system (LCMS-8040) with an electrospray source, coupled to a Shimadzu LC system (Tokyo, Japan). Separation was performed in an Inertsil ODS-3 (C18, 5 μm) 2.1 mm i.d. × 150 mm with a guard cartridge, 2.1 mm i.d. × 10 mm (GL Science Inc., Tokyo, Japan), maintained at 40 °C and eluted at a mobile phase flow rate of 0.2 mL/min. The isocratic mobile phase consisted of 5 mM ammonium acetate and acetonitrile (50:50 v/v). The MS conditions were: electrospray ionization, positive mode; capillary voltage, 4.5 kV; nebulizer gas, nitrogen at a flow rate of 1.5 L/min; drying gas, nitrogen at a flow rate of 15 L/min; vaporizer temperature, 400 °C; and ion transfer capillary temperature, 300 °C. The scan time was set at 0.3 s per transition. The selected reaction monitoring transitions were *m/z* 486.1 to 371.1 for afatinib and *m/z* 446.9 to 128.1 for the internal standard gefitinib, and these were determined by scan mode and reference [13, 14]. Standard curves were linear (r^2^ > 0.99) over the range of 1–600 ng/mL. The lower limit of quantification (LLOQ) of the method was 1 ng/mL. The extraction recovery for afatinib in plasma at 50 ng/mL was 80.62%. For detection of afatinib in plasma samples, the extraction recovery of afatinib at 1, 3, 300, and 480 ng/mL was found to be in the range of 74.47–84.52%. The intra- and inter-batch precisions (RSD %) and the intra- and inter-batch accuracies were within 15%.

### Sample preparation

Plasma samples were separated from blood treated with anticoagulant by centrifugation at 3000×g for 10 min. The obtained plasma was then deproteinized using an equal volume of acetonitrile and centrifuged at 15,000×g for 15 min. An equal volume of gefitinib (0.1 μM) acetonitrile solution as internal standard was subsequently added to the supernatant and centrifuged at 15,000×g for 15 min. Plasma samples were stable for at least 6 months at − 30 °C.

### Experimental outcomes

The primary experimental outcome is to improve skin damage, eliminate scabs and reduce TEWL levels as a result of minocycline ointment. The secondary outcome is microscopic improvement of skin tissue by HE staining.

### Statistical analysis

Pharmacokinetic parameters were evaluated using analysis of variance (ANOVA). Probability values less than 0.05 were considered significant. Statistical analysis was performed with SPSS statistical software (SPSS Inc. Chicago, IL, USA). All parameter values are expressed as means ± SD.

## Results

### Confirmation of rash formation caused by afatinib applied directly to mouse skin

To assess whether minocycline improved the rash caused by afatinib, the transpiration rate of the skin in the presence or absence of the rash was compared in mice. First, afatinib (0.075 mg/0.1 g or 0.1 mg/0.1 g) in white petrolatum (WP) was directly applied to the skin to confirm whether the rash was caused by afatinib. In the group to which the afatinib low concentration (0.75 mg/g) of WP ointment was applied, a macroscopically obvious skin sore was observed after 4 days, and significant skin damage was also observed based on the TEWL. In the 1 mg/g WP ointment application group, more obvious skin damage occurred, and some scab formation was observed. There was no change in weight gain in both groups compared to control group.

### Ameliorating effect of minocycline on the rash caused by directly applied afatinib

**Fig. 1:**
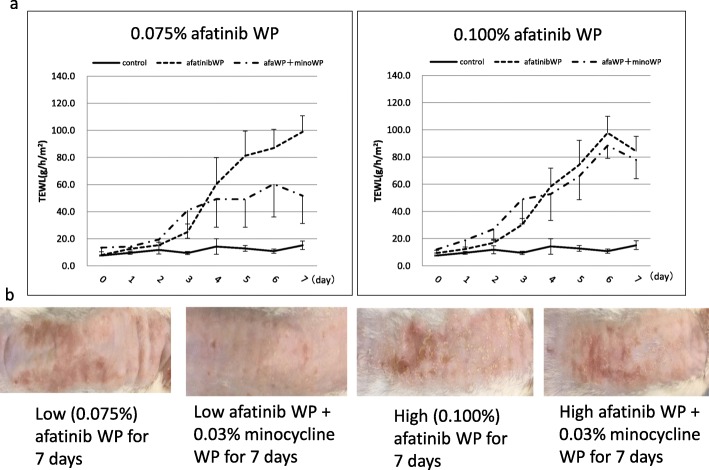
Effects of minocycline WP on rash induced by afatinib ointment application. **a** Left side is the TEWL value of control mice with low concentration (0.075%) afatinib WP for 7 days with/without minocycline WP treatment. Right side is high concentration (0.10%) afatinib WP treatment. **b** Representative images of the effects of treatment with minocycline WP after onset of the rash. Left side is low concentration (0.075%) afatinib WP, and right side is treatment with minocycline WP from 7 days after the start of administration. Data (mean ± SD, *n* = 4 per group) are compared by one-way analysis of variance

The combined application of minocycline ointment (0.3 mg/g) prevented and improved the skin disorder in the afatinib (0.75 mg/g WP) group. A significant difference was also seen in TEWL, suggesting that minocycline improved the skin disorder (Fig. 1). From this result, it was decided that the concentration of minocycline applied to the skin was 0.3 mg/g at a dose of 0.1 g per mouse.

### Ameliorating effect of minocycline on mouse skin rash caused by oral afatinib

It took more than 1 week to develop a skin disorder with oral administration of afatinib. There was no change in weight gain in all groups compared to control, on the other hand, individual differences were large in the onset of skin disorders. The dose of afatinib was 10 or 20 mg/kg BW, and the concentration was set to 0.6 mL/30 g BW of the aqueous solution once a day. At both concentrations, harder rough skin and an increase in TEWL were observed on the neck 10 days after the start of administration. The dose was determined to be 20 mg/kg, since a significant (< 0.01) increase in the TEWL was detected at 20 mg/kg (data not shown).

**Fig. 2:**
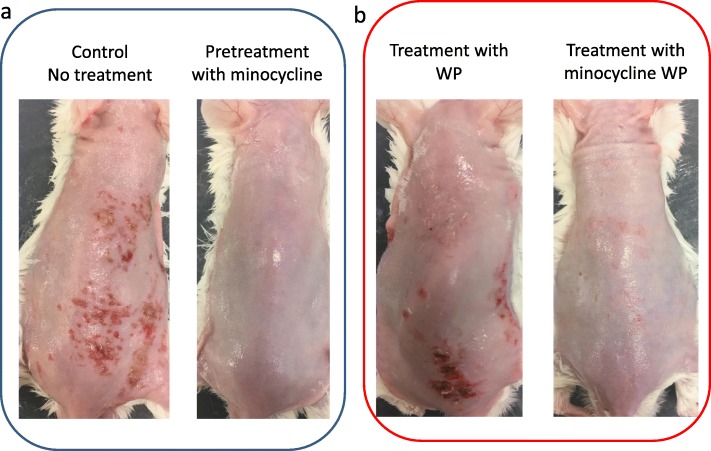
Effects of minocycline on rash induced by afatinib oral administration (20 mg/kg BW). **a** Left side is a representative image of control mice with continued administration for 18 days with no treatment. Right side is a representative image of mice pretreated with minocycline WP from 3 days before afatinib administration. **b** Effects of treatment by WP or minocycline WP after onset of the rash. Left side is treatment with WP, and right side is treatment with minocycline WP from 7 days after the start of administration

**Fig. 3:**
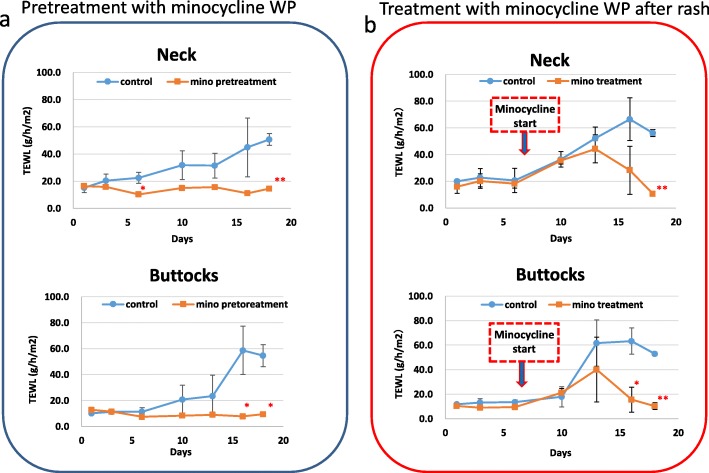
Effect of minocycline WP on the TEWL value after afatinib oral administration. **a** Upper half is the TEWL value of the neck of the mice for 18 days. Lower half is the TEWL value of the buttocks of mice pretreated with minocycline WP (orange square) from 3 days before afatinib administration compared with WP (blue circle) as control. **b** Effects of treatment by WP or minocycline WP after onset of the rash. Upper is neck, and lower is treatment with minocycline WP from 7 days after start of administration. Data (mean ± SD, *n* = 4 per group are compared by one-way analysis of variance. *P < 0.05, **P < 0.01 versus control

In order to clarify the skin damage prevention effect of minocycline WP, after pretreatment of mice with minocycline WP (0.03 mg/0.1 g) for 3 days, oral administration of afatinib did not cause skin damage, and the skin remained in a healthy condition. In addition, when the minocycline WP (0.03 mg/0.1 g) was applied to the skin disorder caused by afatinib, a significant improvement in water loss and macroscopic repair of the skin disorder were observed (Figs. 2 and 3). In all conditions, the concentration of afatinib in plasma and skin was below the limit of quantification.

### Histological changes of the skin on hematoxylin-eosin **(**HE) staining

To elucidate the mechanism of skin symptom improvement by minocycline, histological changes such as keratinocyte arrangement and crust formation were observed. On HE staining, minocycline pretreatment (applied 3 days before afatinib administration) maintained the same condition as the skin of normal mice, and no rash due to afatinib developed. In contrast, in mice treated with afatinib for 21 days, thickening of the epidermis was observed even when no eruption occurred.

**Fig. 4:**
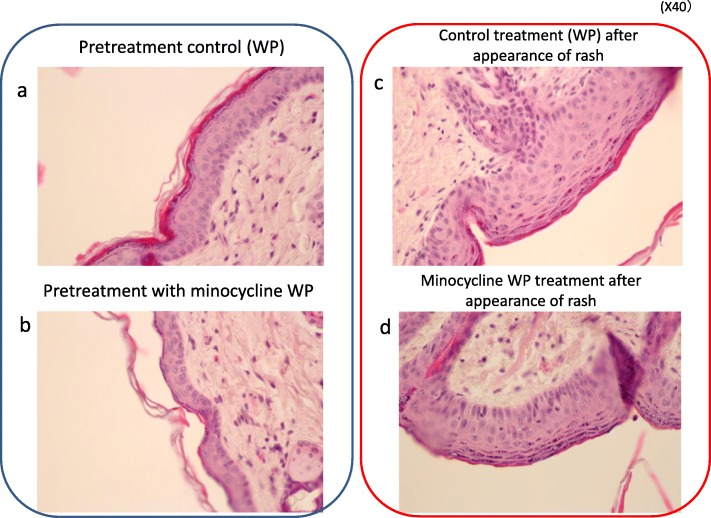
Histological examination by HE staining. **a** pretreatment control with vehicle (:WP), **b** pretreatment with minocycline WP, **c** control treatment with vehicle (:WP) after appearance of rash, and **d** minocycline WP treatment after appearance of rash

**Fig. 5:**
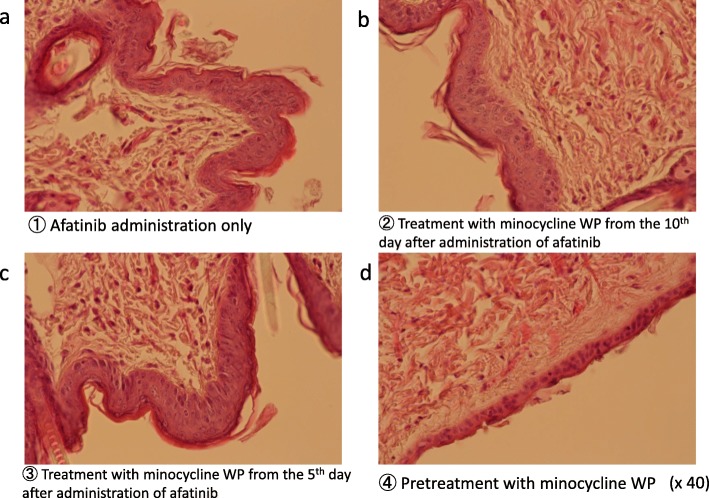
Effect of minocycline WP on histological change after afatinib administration, HE staining. **a** afatinib administration only, **b** treatment with minocycline WP from the 10th day after administration of afatinib, **c** treatment with minocycline WP from the 5th day after administration of afatinib, and **d** pretreatment with minocycline WP before administration of afatinib for 3 days

In the group that received minocycline for the purpose of treatment after appearance of the rash, alignment of keratinocytes was observed about 3–5 days after application, and the disorder gradually recovered (Figs. 4 and 5).

### Effect of minocycline on immunohistological changes of EGFR after afatinib administration

**Fig. 6:**
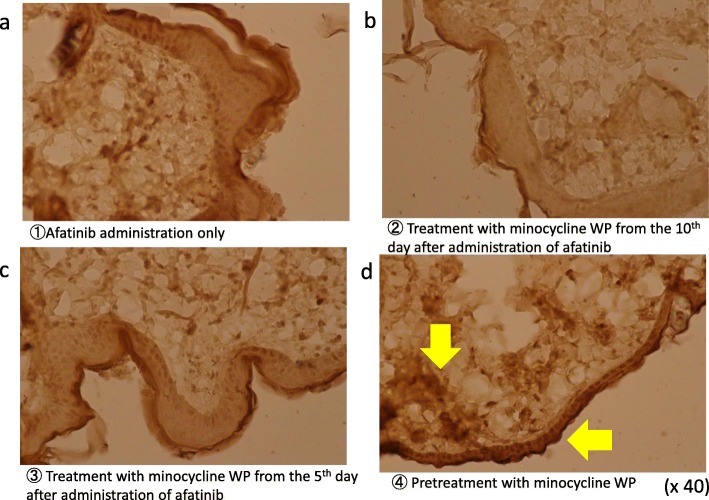
Effect of minocycline WP on immunohistological changes of EGFR after afatinib administration. **a** afatinib administration only, **b** treatment with minocycline WP from the 10th day after administration of afatinib, **c** treatment with minocycline WP from the 5th day after administration of afatinib, and **d** pretreatment with minocycline WP before administration of afatinib for 3 days

Although EGFR appeared to be evenly present on keratinocytes in the skin of normal animals, EGFR antibody staining was decreased in the afatinib-treated group, suggesting an apparent reduction in EGFR (Fig. 6 a-c). Similar to the result of HE staining, in the immunohistochemistry with the EGFR antibody, the group treated with minocycline WP (applied 3 days before afatinib administration) maintained the same state as the skin of normal mice, and showed high concentrations in the epidermis and hair follicles. High EGFR regions were indicated by arrows (Fig. 6-d).

## Discussion

In the present study, (1) a model of drug-induced rash was developed in mice by applying afatinib ointment, and the skin repair effect of minocycline ointment on the afatinib-induced skin disorder was demonstrated, and (2) oral administration of afatinib caused drug-induced skin eruption in mice, and minocycline application exerted a skin repair effect on the afatinib-induced skin disorder. This experiment began with the question of whether current treatments for afatinib-induced skin disorders were appropriate. Our results suggested the topical treatment with direct minocycline application for the skin disorders caused by orally administered afatinib and proved its efficacy. In this study, it is not clear whether minocycline acts directly on EGFR or whether it improves secondary skin damage by the ability of minocycline to repair tissues. We are currently investigating the expression of EGFR in the epidermis and factors or cytokines that are affected by minocycline WP treatment. In the process, we came across the following report.

The EGFR plays an important role in tissue homeostasis and tumor progression. However, cancer patients treated with EGFR inhibitors (EGFRIs) frequently develop acneiform skin toxicities, which are a strong predictor of a patient’s treatment response [15]. Their findings demonstrated that EGFR signaling in keratinocytes regulates key factors involved in skin inflammation, barrier function, and innate host defense, providing insights into the mechanisms underlying EGFRI-induced skin pathologies [15]. In this study Lichtenberger et al., showed that the early inflammatory infiltrate of the skin rash induced by EGFRI is dominated by dendritic cells, macrophages, granulocytes, mast cells, and T cells. EGFRIs induce expression of chemokines (CCL2, CCL5, CCL27, and CXCL14) in epidermal keratinocytes and impair the production of antimicrobial peptides and skin barrier proteins. Correspondingly, EGFRI-treated keratinocytes facilitate lymphocyte recruitment, but they show a considerably reduced cytotoxic activity against *Staphylococcus aureus*. From these results, it was considered that the cause of skin damage induced by EGFR inhibitors was CCL27, especially chemokines. Furthermore, due to the dysfunction of EGFR, the release of cytokines such as IL-6 and TNF-α and the decreased production of cell adhesion factors such as claudin resulted in dry skin. It was also shown that growth was suppressed, and the average survival time was shortened. Mice lacking epidermal EGFR (EGFRΔep) show a similar phenotype, which is accompanied by chemokine-driven skin inflammation, hair follicle degeneration, decreased host defense, and deficient skin barrier function, as well as early lethality [15].

On the other hand, pharmacokinetic studies have shown that EGFR-TKIs are transported to the skin by ABCG2 (BCRP) and/or ABCC (P-gp) [16, 17]. The problem is how inhibition of EGFR causes skin damage. Ashida et al. suggested a mechanism by which inflammation is expressed by increasing the amount of TNF-α due to activation of the PPARr-COX-2 pathway by EGFR inhibition and activation of NF-kB in sebocytes. These results suggest that inhibiting the PPARr-COX-2 and NF-kB pathways may be a possible treatment method that reduces the skin damage caused by EGFRIs [18]. Another study reported that the failure of EGFR homeostasis resulted in abnormal differentiation of sebocytes and the formation of acne-like eruptions [19], but there is still no clear explanation for the mechanism.

The beginning of our interest in minocycline was Torigoe’s report that intrathecal administration of minocycline suppressed itch in atopic dermatitis and improved dermatitis. Based on this report, the experiment using this mouse model was planned and implemented [9]. In addition, it has been reported that minocycline acts on a mitochondrial protein and is involved in preventing the onset of Parkinson’s disease (PD) [10]. Furthermore, minocycline has been attracting attention for its action on nerve cells; for example, it is expected to suppress the risk of developing multiple sclerosis [11]. Thus, since minocycline is a very interesting drug, further research should proceed in many clinical fields.

The limitation of our study is that the number of animal experiments is small. In our experiment examining the effect of minocycline ointment on afatinib-induced skin disorders (Fig. 3), a significant difference of less than 0.01 was observed in TEWL value on Day 18, but no significant difference was found on Day 15 depending on the part of the back. These results were considered to be due to individual differences in the appearance of afatinib-induced rash and skin repair ability. We are continuing to confirm the reproducibility of the effects of minocycline ointment on skin disorders. Further, it is necessary to carry out experiments such as verifying the effects of minocycline administered orally, comparing the effects of other tetracyclines, and comparing the effects with corticosteroids. Based on these results, it is required to conduct clinical tests on patients using EGFR-TKI, including patients with non-small cell lung cancer, as soon as possible.

## Conclusion

The present results suggest that application of minocycline is effective for the prevention and repair of skin disorders caused by afatinib, one of EGFR-TKIs. Histological examination of the skin elucidated the mechanism of the skin disorder caused by afatinib and suggested the clinical efficacy of topical minocycline application.

## Data Availability

The datasets used and analyzed in the current study are available from the corresponding author on reasonable requests.

## Abbreviations

EGFR: Epidermal growth factor receptor
TKI: Tyrosine kinase inhibitor
TEWL: Transepidermal water loss
NSCLC: Non-small cell lung cancer
LC/MS: Liquid chromatography-mass spectrometry
HE: Hematoxylin-eosin
ABCG2 (BCRP): ATP-binding cassette sub-family G member 2 (breast cancer resistance protein)
ABCB1 (P-gp): ATP-binding cassette sub-family B member 1 (p-glycoprotein)
